# Supplementary material for: Emergence of charge density waves and a pseudogap in single-layer TiTe2
Source: Nat Commun. 2017 Sep 11;8:516. doi: 10.1038/s41467-017-00641-1 (PMC5593837; doi:10.1038/s41467-017-00641-1)
Supplement: Supplementary file 1 — Supplementary Information [file 41467_2017_641_MOESM1_ESM.pdf]

Description of Supplementary Files

File Name: Supplementary Information
Description: Supplementary Figures, Supplementary Notes and Supplementary References

File Name: Peer Review File

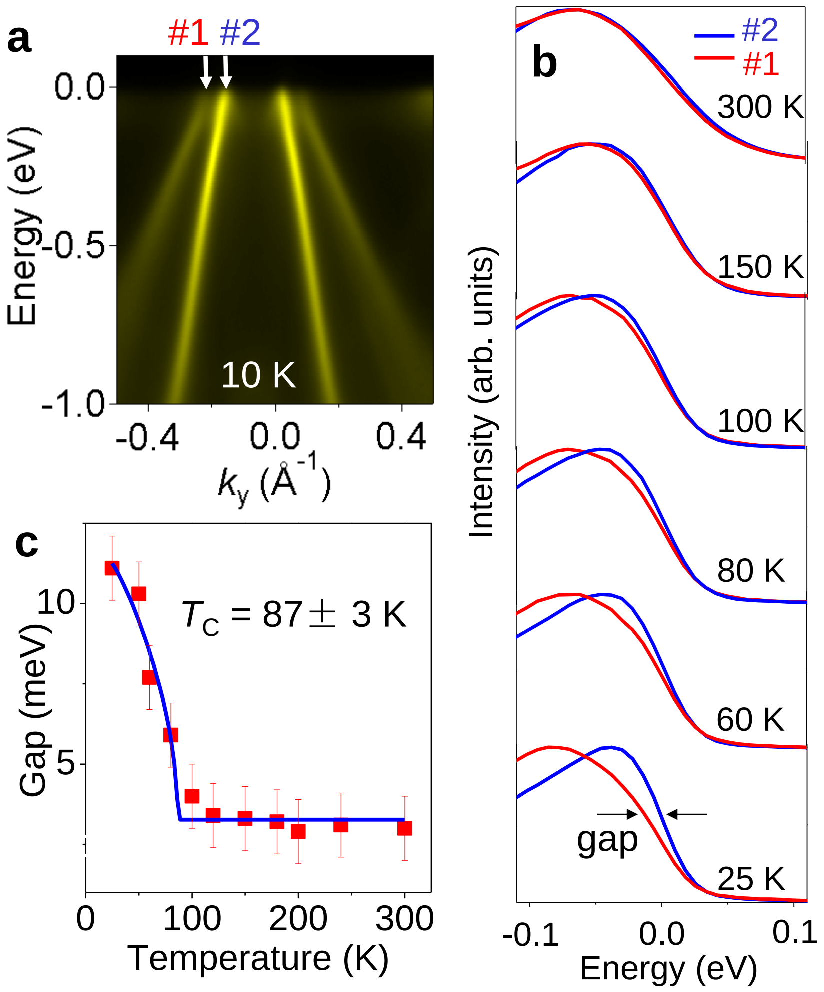

Supplementary Figure 1. ARPES spectra on valence bands and gap analysis. a, ARPES map around the zone center. Two valence bands (#1 and #2) cross the Fermi level at points indicated by the arrows. **b,** Energy distribution curves at the two *k* points indicated in (**a**) for various sample temperatures as indicated. At lower temperatures, the edges sharpen, but the red EDC (#1 cut) recedes from the edge relative the blue EDC (#2 cut), resulting in a gap as indicated for the 25 K case. **c,** The gap associated with band #1 as a function of temperature. The blue curve is a mean-field fit. The resulting T_C = 87 ± 3 K for the ARPES gap agrees well with the value of 92 ± 3 K based on the analysis of the folded-band intensity.

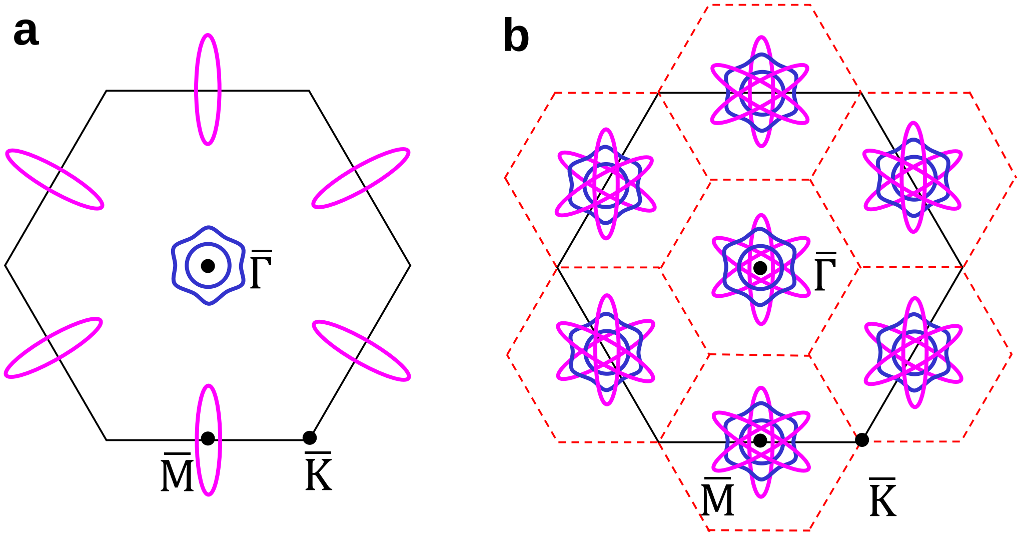

Supplementary Figure 2. Calculated Fermi surface with 1x1 and 2x2 structure. a, Calculated Fermi surfaces of undistorted single-layer $TiTe_2$. b, The same Fermi surfaces after 2x2 folding.

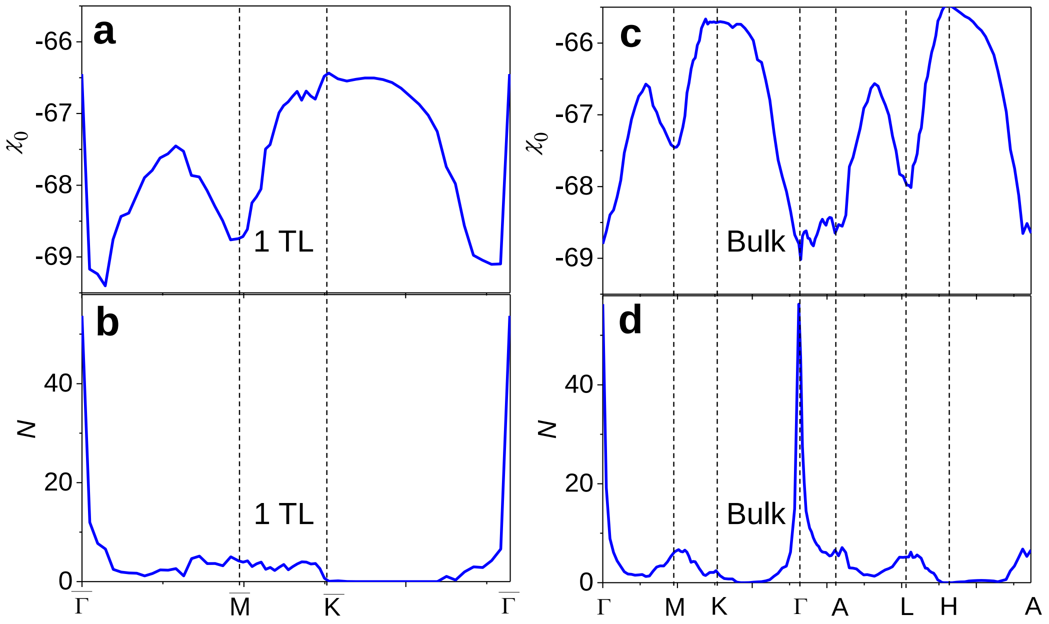

Supplementary Figure 3. Calculated electronic susceptibility and nesting function to further check the electronic instability. Computed electronic susceptibility χ_0 and nesting function N for single-layer and bulk $TiTe_2$ as indicated.

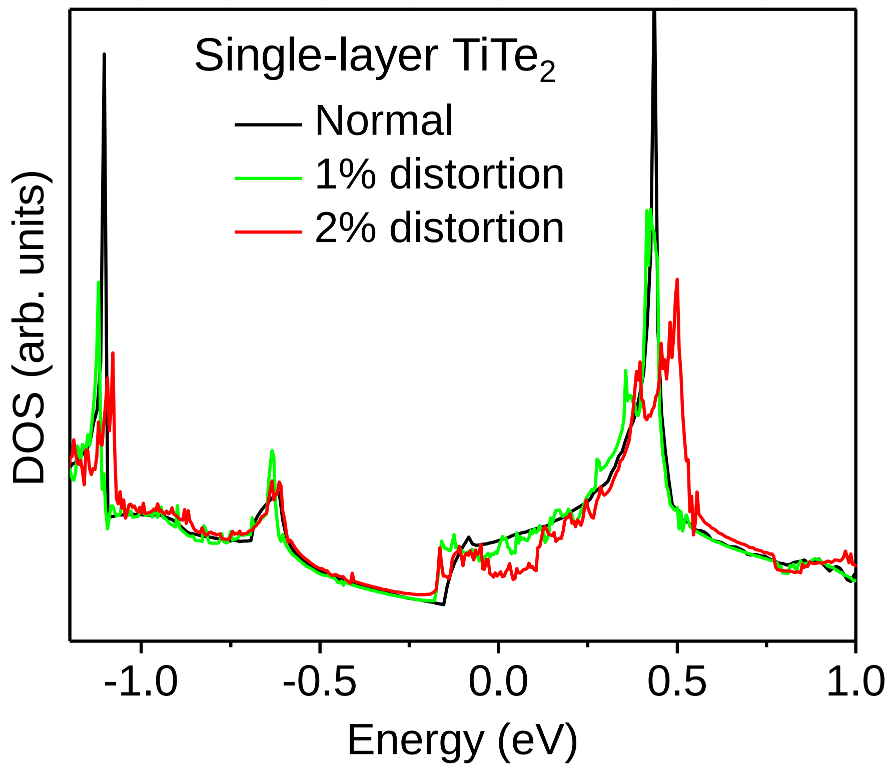

Supplementary Figure 4. Calculated density of states with different Ti atomic displacements. Computed density of states (DOS) for single-layer $TiTe_2$ with a CDW distortion pattern imposed on the lattice with a fixed lattice constant, assuming an amplitude of the Ti atomic displacement equal to 0%, 1%, or 2% of the lattice constant as indicated.

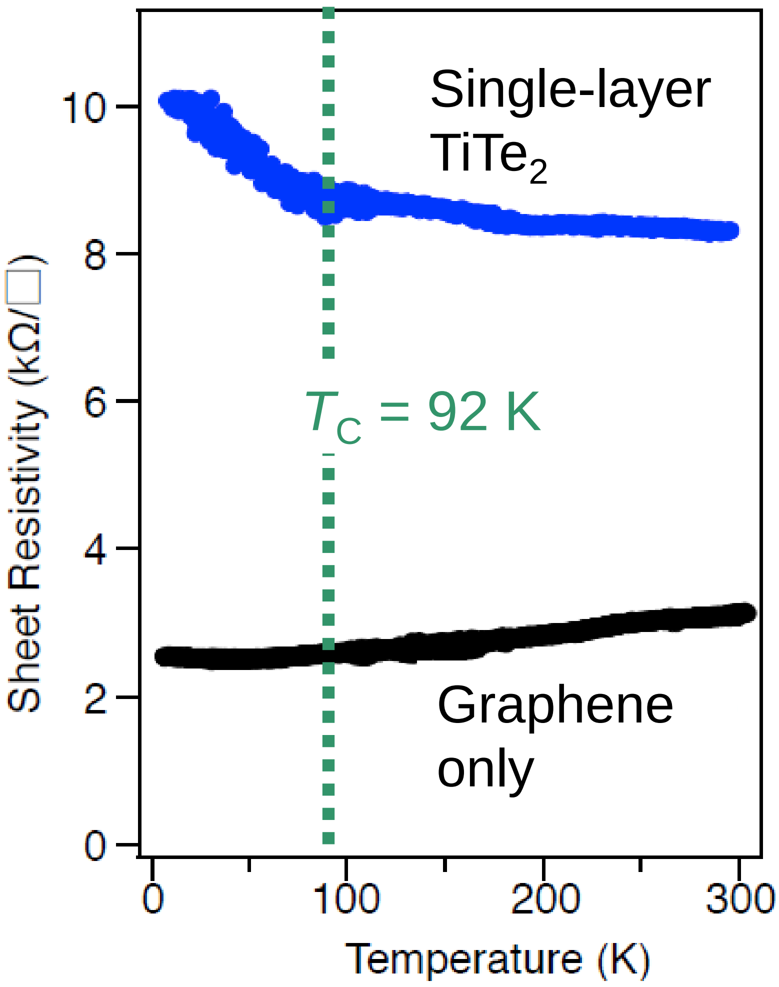

Supplementary Figure 5. Sheet resistance of single-layer $TiTe_2$ and substrate. Temperature dependence of the sheet resistance of single-layer $TiTe_2$ in the temperature range of 10-300 K. Also shown are results taken from the substrate without $TiTe_2$ for comparison. The CDW transition temperature of 92 K from ARPES measurement is indicated with a vertical dashed line.

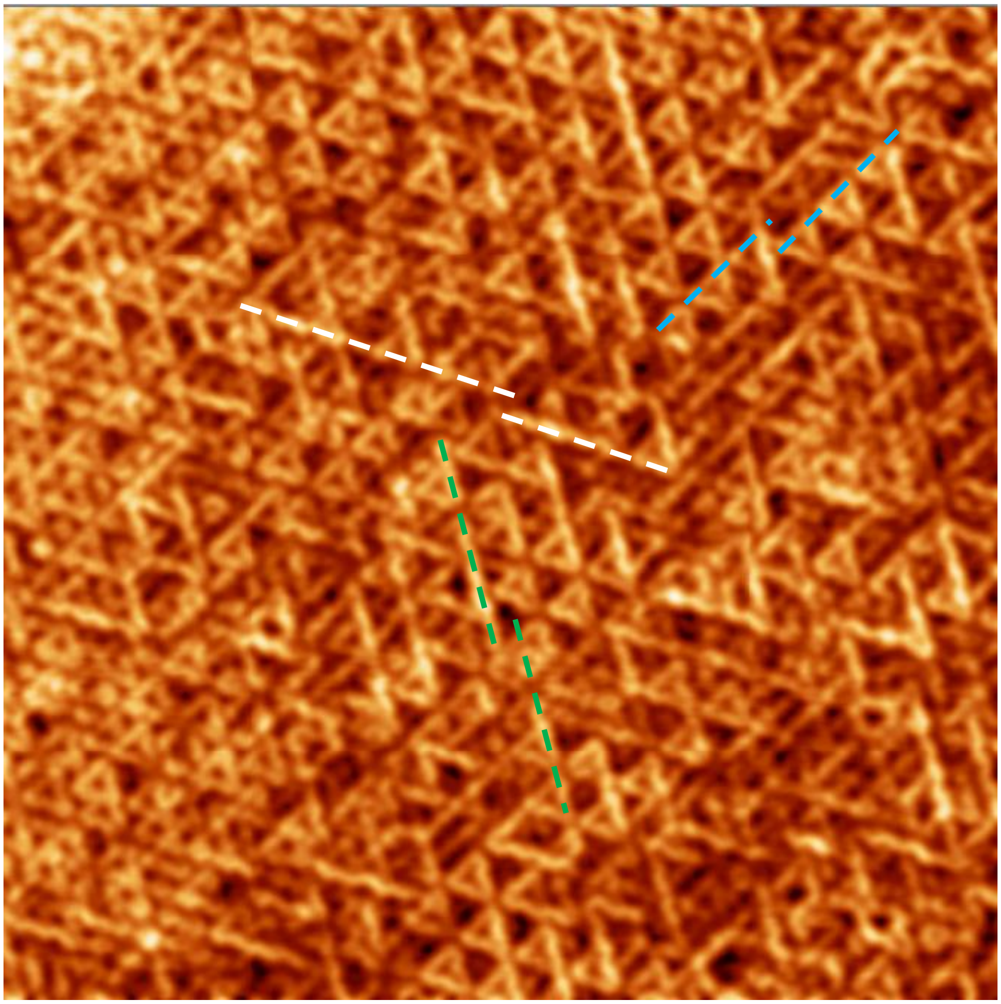

Supplementary Figure 6. CDW domains in single-layer $TiTe_2$. This image is created from Fig. 1 in the main text by merging the two derivative images in the X and Y directions, followed by slight Gaussian smoothing. The three sets of colored lines are oriented along symmetry-equivalent close-packed directions. In each set, the two lines are drawn along close packed chains of atoms on the surface in two neighboring domains. The offset corresponds to a (1x1) unit cell translation, or one half of a (2x2) unit cell translation. This half translation gives rise to a domain boundary at the closest point of the two lines.

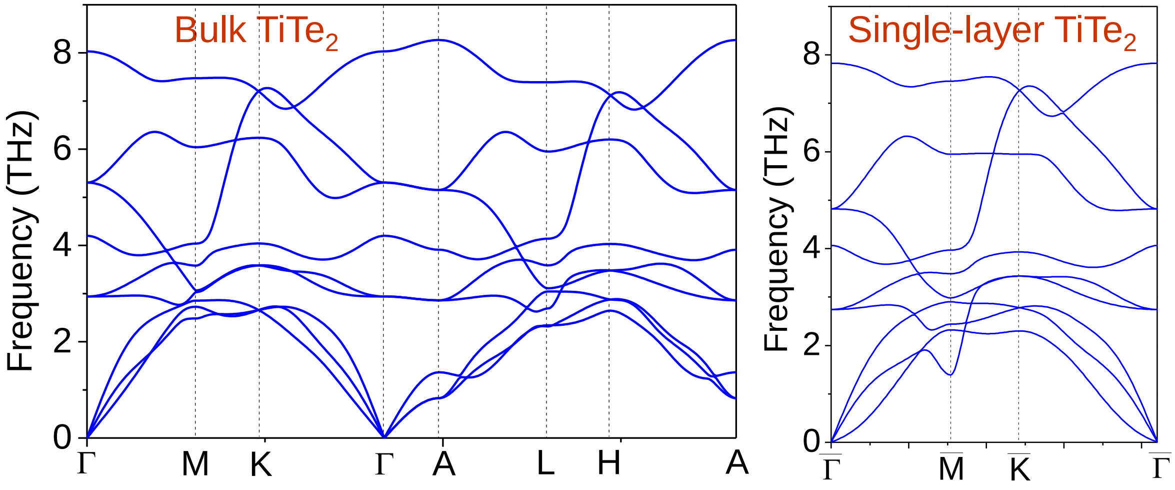

Supplementary Figure 7. Calculated phonon dispersions. Calculated phonon dispersion relations for bulk and single-layer $TiTe_2$.

Supplementary Note 1. ARPES evidence for a pseudogap

The STS spectrum (Fig. 4) shows a pseudogap. One would expect that ARPES spectra should also show evidence for a gap. Unlike STS which contains spectral information integrated over a large area in the two-dimensional Brillouin zone, ARPES yields *k*-resolved spectral functions. A direct comparison between the two measurements is not easily feasible. Instead, we make vertical cuts in the ARPES map in Supplementary Fig. 1a at the points in *k* space where the two valence bands (#1 and #2) cut through the Fermi level. The resulting energy distribution curves (EDCs) in Supplementary Fig. 1b, after proper intensity normalization for easy comparison, show essentially the same shape at high temperatures. At lower temperatures, both become narrower because of reduced thermal broadening, but the red EDC (#1 cut) recedes from the edge relative the blue EDC (#2 cut). If we define an effective pseudogap as given by twice the difference between the binding energies of the leading-edge midpoints between the two cases [1,2], the gap is 22 meV at 25 K. This is fairly close to the 28 meV gap measured by STS at 4.2 K. While this comparison is not rigorous, we conclude that ARPES does reveal a gap-like effect with a similar magnitude. Evidently, band #1 (the topmost valence band) is the one that develops a gap. If the spectral contributions from both bands are considered, this would give rise to a partial gap, or a pseudogap, as observed in STS.

The temperature dependence of the ARPES gap of band #1 (points in Supplementary Fig. 1c) can be well fitted by the mean-field gap equation:

$$\Delta(T) \propto \tanh\left(A\sqrt{\frac{T_C}{T} - 1} \right) \Theta(T_C - T) \quad (1)$$

as indicated by the blue curve. For this fit, we use the same value of $A = 1.37$ deduced from the analysis for the folded-band intensity in Fig. 2g. The resulting $T_C = 87 \pm 3$ K for the ARPES gap

agrees well with the value of 92 ± 3 K based on the analysis of the folded-band intensity. This agreement indicates that the ARPES gap is indeed associated with the CDW transition.

Supplementary Note 2. Question about Fermi surface nesting

The calculated Fermi surfaces of single-layer $TiTe_2$ (Supplementary Fig. 2a) show two hole pockets centered at $\bar{\Gamma}$ and six electron pockets centered at $\bar{\mathrm{M}}$. The same Fermi surfaces after folding into (2x2) zones (Supplementary Fig. 2b) show no evidence of any (2x2) nesting; instead, the Fermi contours associated with electron and hole pockets intersect at discrete points.

Supplementary Note 3. Nesting function and electronic susceptibility

We have computed the nesting function of the system as a further check of any tendency for the system to undergo a CDW transition [3]. This function is defined by

$$N(\mathbf{q}) = \frac{1}{N_{\mathbf{k}}} \sum_{m,n,\mathbf{k}} \delta\left(\varepsilon_{n,\mathbf{k}}\right) \delta\left(\varepsilon_{m,\mathbf{k}-\mathbf{q}}\right) \quad (2)$$

where n and m are band indices, $N_{\mathbf{k}}$ is the number of **k** points in the summation, and $\varepsilon_{n,\mathbf{k}}$ is the band energy with the Fermi level set to zero. It is a measure of the joint density of states at the Fermi energy; nesting at a wave vector **q** would lead to a strong sharp peak. The results for bulk $TiTe_2$ (Supplementary Fig. 3d) show very small humps at the M and L points in the Brillouin zone, which could indicate a very slight tendency toward (2x2x1) or (2x2x2) ordering. However, we know that bulk $TiTe_2$ does not exhibit a CDW transition. By comparison, the nesting function for the single layer (Supplementary Fig. 3b) is essentially featureless. The results suggest that nesting cannot be a reason for the (2x2) ordering in the single layer.

Another quantity of interest is the static bare electronic susceptibility χ_0; strong features in this function may indicate electronic instability [4, 5]. This function is also connected to the phonon self energy [5]; a minimum indicates phonon softening. The function χ_0 is defined by

$$\chi_0(\mathbf{q}) = \frac{1}{N_{\mathbf{k}}} \sum_{m,n,\mathbf{k}} \frac{f_{n,\mathbf{k}} - f_{m,\mathbf{k-q}}}{\varepsilon_{n,\mathbf{k}} - \varepsilon_{m,\mathbf{k-q}}} \qquad (3)$$

where $f_{n,\mathbf{k}}$ is the occupation function. Calculations for bulk $TiTe_2$ at $T = 0$ (Supplementary Fig 3c) show very weak and broad negative peaks (at the few percent level) at M and L. The results for the single layer (Supplementary Fig. 3a) also show a very weak and broad negative peak at $\bar{\mathrm{M}}$. These features are too weak to be of any real consequences, and we know that bulk $TiTe_2$ does not exhibit a CDW transition. We conclude that the CDW in the single layer cannot be explained by the conventional arguments based on the electronic susceptibility and the nesting function.

Supplementary Note 4. Density of states (DOS) for distorted structures of single-layer $TiTe_2$

For a further check of the origin of the pseudogap seen by STS, we have computed the electronic DOS for single-layer $TiTe_2$ assuming a distorted pattern of the lowest phonon mode at the $\bar{\mathrm{M}}$ point. The same phonon mode is responsible for the CDW in $TiSe_2$. The distortion pattern is imposed on the lattice with a fixed lattice constant, assuming an amplitude of the Ti atomic displacement equal to 1% or 2% of the lattice constant. The resulting DOS functions, including that for the undistorted lattice, are shown in Supplementary Fig. 4. At 1% distortion, the DOS function does not change very much. At 2% distortion, a gap-like feature is observed near the Fermi level. It can be attributed to the hybridization of the states near the Fermi level where the electron and hole pockets cross under the (2x2) distortion. However, the computed gap for the assumed structure, about 0.2 eV, is much wider than the STS gap. It is not symmetric about the

Fermi level and does not go down as far as the STS gap. This simulation again rules out Fermi surface nesting as the mechanism for the CDW in the single layer.

Supplementary Note 5. Sheet resistance of single-layer $TiTe_2$

Electronic transport measurements were performed with a UHV micro-four-point probe system at temperatures ranging from room temperature to 10 K. The four probes are made of copper wires, and the probe spacing is 100 μm. The apparatus was also equipped with a RHEED system for sample characterization. The sheet resistance was obtained by a DC current-voltage measurement, using the dual configuration method to avoid data scattering caused by variations of the probe contact point. [6]

To avoid conduction within the substrate, we used undoped SiC to prepare the $TiTe_2$ films for the measurement. Supplementary Figure 5 shows the temperature dependence of the sheet resistance for single-layer $TiTe_2$. Upon decreasing temperature, it shows a break or an onset at ~90 K, below which the resistance increases substantially. The behavior suggests a gap opening. These results agree with the CDW transition at 92 K as observed by ARPES and the pseudogap revealed by STS and ARPES.

Supplementary Note 6. CDW domains in single-layer $TiTe_2$

The observed CDW in single-layer $TiTe_2$ is rather weak, in contrast to the much stronger CDW in, e.g., $TiSe_2$. It's not unexpected that the very weak $TiTe_2$ CDW can be affected by random perturbations caused by defects and impurities in the substrate and the film. With a (2x2) superstructure, random pinning can give rise to antiphase domains; the domain boundaries become topological defects. Specifically, an antiphase domain is formed if a portion of the original lattice

is shifted by one unit vector. This type of defects is common in systems with multiple atoms in a unit cell. For an illustration of the domain structure, we show in Supplementary Fig. 6 a processed image derived from the STM image shown in Fig. 1. It is obtained by merging the two derivative images in the *X* and *Y* directions, followed by slight Gaussian smoothing. The three sets of colored lines in Supplementary Fig. 6 are oriented along symmetry-equivalent close-packed directions. In each set, the two lines are drawn along close packed chains of atoms on the surface in two neighboring domains. The offset corresponds to a (1x1) unit cell translation, or one half of a (2x2) unit cell translation. This half translation gives rise to a domain boundary at the closest point of the two lines.

Supplementary Note 7. Electron-phonon coupling and calculated phonon dispersion relations in bulk and single-layer $TiTe_2$

It is interesting to make a comparison with $NbSe_2$, for which the CDW transition temperature of 33 K in the bulk becomes 145 K in a single layer; the results possibly suggest an enhanced electron-phonon coupling in the single-layer leading to the ~4x increase of the transition temperature [7]. Another case of interest is $TiSe_2$, which has an electronic structure resembling that of $TiTe_2$ (while the electronic structure of $NbSe_2$ looks very different). $TiSe_2$ exhibits a CDW transition at 205 K in the bulk and 232 K in a single layer [8]. The interpretation there is different; each layer forms (2x2) first, and then at a lower temperature the layers phase lock and freeze into the bulk (2x2x2) CDW structure. Both interpretations are possibilities for $TiTe_2$, but it is hard to prove one way or the other. As pointed out in the main text, the $TiTe_2$ case is unusual and mysterious because bulk $TiTe_2$, unlike $NbSe_2$ and $TiSe_2$, does not exhibit a CDW transition. In fact, no CDW transition is observed even at just two layers of $TiTe_2$. The 92 K transition in single-

layer $TiTe_2$, accompanied by a pseudogap that cannot be explained by any of the known theories, points to possibly unconventional mechanisms.

Another related issue of interest in that superconductivity in bulk $TiTe_2$ has not been detected down to a temperature of 0.45 K at ambient pressure [9]. The lack of a superconducting transition and a CDW transition in the bulk may imply weak electron-phonon coupling effects in this system. To look for further clues, we have computed the phonon dispersion relations for a (1x1) single layer and bulk $TiTe_2$, which are shown in Supplementary Fig. 7. There is nothing unusual about the bulk dispersion relations. The small dip in an acoustic branch at $\bar{\mathrm{M}}$ for the single layer might suggest a tendency for (2x2) distortion, but there are no imaginary frequencies that would correspond to structural instabilities. Within our numerical accuracy, we do not find a (2x2) transition. Additional phonon calculations were performed at the slightly different experimental lattice constant. The differences are very slight; so, there are no surprises. Other effects including spin-orbit coupling and Hubbard U have also been tested. No phonon modes with imaginary frequencies are ever observed.

Supplementary References

1. Borisenko, S. V. *et al.*, Pseudogap and charge density waves in two dimensions. *Phys. Rev. Lett*. **100**, 196402 (2008).

2. Borisenko, S. V. *et al.*, Two Energy Gaps and Fermi-Surface “Arcs” in $NbSe_2$. *Phys. Rev. Lett*. **102**, 166402 (2009)

3. Calandra, M. & Mauri, F. Charge-Density wave and superconducting dome in $TiSe_2$ from electron-phonon interaction, *Phys. Rev. Lett.* **106**, 196406 (2011).

4. Johannes, M. D., Mazin, I. I. & Howells, C. A. Fermi-surface nesting and the origin of the charge-density wave in $NbSe_2$, *Phys. Rev. B* **73**, 205102 (2006).

5. Calandra, M., Mazin, I. I. & Mauri, F. Effect of dimensionality on the charge-density wave in few-layer 2H-$NbSe_2$, *Phys. Rev. B* **80**, 241108 (2009).

6. Yamada, M *et al.*, Surface electrical conductivity measurement system with micro-four-point probes at sub-Kelvin temperature under high magnetic field in ultrahigh vacuum. e-J.Surf. Sci. Nanotechnol. **10**, 400−405 (2012).

7. Xi, X., Zhao, L., Wang, Z., Berger, H., ForrÓ, L., Shan, J. & Mak, K., Strongly enhanced charge-density-wave order in monolayer $NbSe_2$. *Nature Nanotech.* **10**, 765–769 (2015).

8. Chen, P., Chan, Y.-H., Fang, X.-Y., Zhang, Y., Chou, M.Y., Mo, S.-K., Hussain, Z., Fedorov, A.-V. & Chiang, T.-C. Charge density wave transition in single-layer titanium diselenide. *Nat. Commun.* **6**, 8943 (2015).

9. Koike, Y., Okamura, M., Nakanomyo, T. & Fukase, T., Log *T* Dependence of Resistivity and Negative Magnetoresistance in the Layered Compound $TiTe_2$. *J. Phys. Soc. Jpn.* **52**, 597 (1983).
